# Magnetic Resonance Imaging of Mouse Islet Grafts Labeled with Novel Chitosan-Coated Superparamagnetic Iron Oxide Nanoparticles

**DOI:** 10.1371/journal.pone.0062626

**Published:** 2013-04-29

**Authors:** Jyuhn-Huarng Juang, Chia-Rui Shen, Jiun-Jie Wang, Chien-Hung Kuo, Yu-Wen Chien, Hsiao-Yunn Kuo, Fu-Rong Chen, Ming H. Chen, Tzu-Chen Yen, Zei-Tsan Tsai

**Affiliations:** 1 Division of Endocrinology and Metabolism, Department of Internal Medicine, Chang Gung Memorial Hospital and Department of Medicine, College of Medicine, Chang Gung University, Taoyuan, Taiwan; 2 Department of Medical Biotechnology and Laboratory Science, College of Medicine, Chang Gung University, Taoyuan, Taiwan; 3 Molecular Imaging Center, Chang Gung Memorial Hospital, Linkou, Taoyuan, Taiwan; 4 Department of Medical Imaging and Radiological Sciences, College of Medicine, Chang Gung University, Taoyuan, Taiwan; 5 Department of Biological Science and Technology, National Chiao Tung University, Hsinchu, Taiwan; 6 Center of Transmission Microscopy, National Tsing Hua University, Hsinchu, Taiwan; 7 Surgical-Medical Research Institute, University of Alberta, Edmonton, Canada; 8 Department of Nuclear Medicine, Chang Gung Memorial Hospital, Taoyuan, Taiwan; St. Vincent’s Institute, Australia

## Abstract

**Object:**

To better understand the fate of islet isografts and allografts, we utilized a magnetic resonance (MR) imaging technique to monitor mouse islets labeled with a novel MR contrast agent, chitosan-coated superparamagnetic iron oxide (CSPIO) nanoparticles.

**Materials and Methods:**

After being incubated with and without CSPIO (10 µg/ml), C57BL/6 mouse islets were examined under transmission electron microscope (TEM) and their insulin secretion was measured. Cytotoxicity was examined in α (αTC1) and β (NIT-1 and βTC) cell lines as well as islets. C57BL/6 mice were used as donors and inbred C57BL/6 and Balb/c mice were used as recipients of islet transplantation. Three hundred islets were transplanted under the left kidney capsule of each mouse and then MR was performed in the recipients periodically. At the end of study, the islet graft was removed for histology and TEM studies.

**Results:**

After incubation of mouse islets with CSPIO (10 µg/mL), TEM showed CSPIO in endocytotic vesicles of α- and β-cells at 8 h. Incubation with CSPIO did not affect insulin secretion from islets and death rates of αTC1, NIT-1 and βTC cell lines as well as islets. After syngeneic and allogeneic transplantation, grafts of CSPIO-labeled islets were visualized on MR scans as persistent hypointense areas. At 8 weeks after syngeneic transplantation and 31 days after allogeneic transplantation, histology of CSPIO-labeled islet grafts showed colocalized insulin and iron staining in the same areas but the size of allografts decreased with time. TEM with elementary iron mapping demonstrated CSPIO distributed in the cytoplasm of islet cells, which maintained intact ultrastructure.

**Conclusion:**

Our results indicate that after syngeneic and allogeneic transplantation, islets labeled with CSPIO nanoparticles can be effectively and safely imaged by MR.

## Introduction

Patients with type 1 diabetes mellitus are characterized by progressive β-cell destruction which leads to insulin deficiency and eventually insulin dependency [1]. In contrast to insulin therapy, β-cell replacement via pancreas and islet transplantation can precisely adjust the changes in the blood glucose level and is thus more physiologically relevant for the treatment [2]. Human islet transplantation has achieved insulin independence in type 1 diabetes. Its success rate has been markedly improved by the Edmonton Protocol [3], [4]. However, its long-term results are disappointing, only 10% of the recipients maintain insulin independence 5 years post-transplantation [5]. Even though, 80% of them were C-peptide positive, which indicates the existence of grafted β-cells [5]. To better understand the fate of transplanted islets and its relationship with graft function and overall glucose homeostasis, an accurate, reproducible, and noninvasive method of islet imaging is needed [6], [7].

In the past years, a magnetic resonance (MR) imaging technique has been used to detect transplanted islets labeled with dextran-coated superparamagnetic iron oxide (SPIO), such as ferumoxide (Feridex®, Endorem™) and Ferucarbotran (Resovist^®^) in mice [8]–[11], rats [12]–[19], baboons [20], and humans [21], [22]. Unfortunately, the manufacturing of Feridex® and Resovist® was ceased in 2008 and 2009, respectively [23]. Therefore, it is crucial to develop new MR contrast agents for islet imaging. Chitosan is the N-deacetylated product of chitin, which is one of the most abundant polysaccharides in nature. It has been applied to numerous biomedical applications due to its nontoxicity, biocompatibility, and biodegradability [24]. It is particularly interesting in metal nanoparticle synthesis because of its interaction with metal atoms, metal ions, and metal oxide nanoparticles for their stabilization in colloidal suspension. Recently, we developed an in situ coating method for preparing ferrofluids coated with γ-ray irradiated chitosan and proved that the chitosan-coated SPIO (CSPIO) nanoparticles have potential as an MR T2 contrast agent [25]. In addition, we demonstrated that CSPIO nanoparticles could be used for long-term tracking of islet isografts [26] and allografts [27]. In this study, we have further investigated the uptake of CSPIO by isolated islets, the influence of CSPIO on insulin secretion and cell death of islets, and correlations between MR images and histological findings and electromicroscopic studies of CSPIO-labeled islet grafts.

## Materials and Methods

### Ethics Statement

All protocols using animals in this study were approved by the Institutional Animal Care and Use Committee of Chang Gung Memorial Hospital, Taoyuan, Taiwan (IACUC 2008061902).

### Animals

Animals were purchased from the National Laboratory Animal Center, Taipei, Taiwan. Male C57BL/6 mice aged 8–12 weeks were used as donors and 8–12-week-old male inbred C57BL/6 and male Balb/c mice were used as recipients of islet transplantation. The diabetic recipients were made by a single intraperitoneal injection of streptozotocin (STZ, Sigma Immunochemicals, St. Louis, MO, USA, 200 mg/kg body weight, freshly dissolved in citrate buffer, pH 4.5).

### Islet Isolation

Under anesthesia with sodium amobarbital, C57BL/6 mouse pancreases were distended with 2.5 mL of RPMI-1640 medium (GIBCO BRL, Grand Island, NY, USA) containing 1.5 mg/mL of collagenase (collagenase from *Clostridium histolyticum*, type XI, Sigma Immunochemicals, St Louis, Mo, USA), excised and incubated in a water bath at 37°C. Islets were separated by a density gradient (Histopaque-1077; Sigma Immunochemicals), and purified islets were then handpicked under a dissecting microscope [26], [27].

### Islet Labeling

Isolated C57BL/6 mouse islets were incubated overnight in the culture medium containing10 µg/mL CSPIO (Molecular Imaging Center, Chang Gung Memorial Hospital, Taoyuan, Taiwan). After overnight incubation at 37°C in a 5% CO2 atmosphere, islets were washed with culture medium and subsequently used for in vitro studies or islet transplantation [27].

### Insulin Secretion of CSPIO-labeled Islets

The islet secretory response to glucose was determined using static incubation and perifusion assay of labeled and nonlabeled islets at low and high glucose concentrations. After overnight incubation with and without CSPIO, 30 islets were cultured for 120 min in RPMI medium supplemented with 2.8 mmol/L glucose followed by 16.7 mmol/L glucose. Stimulation indices were calculated by dividing the amount of insulin released with 16.7 mmol/L glucose by that released with 2.8 mmol/L glucose [28]. For perifusion study, 150 islets were placed into a perifusion chamber composed of a barrel of 1 ml plastic tuberculin syringe (TERUBO, Tokyo, Japan) and 2 layers of nylon net with 10 µm pores (Pharmacia, Uppsala, Sweden) placed in the bottom. Medium RPMI-1640 gassed with 95% O_2_/5% CO_2_ was pumped through the chamber at a flow rate of 180 µL/min. Islets were first perifused for 2 h with medium containing 5.6 mmol/L glucose to equilibrate the cellular conditions, and then with medium containing 16.7 mmol/L glucose for 30 min. The perifusate was collected at 1-min intervals throughout the experiment, and all the samples were measured by radioimmunoassay [28].

### Cytotoxicity Assay of CSPIO-labeled Islets

The fluorescence of propidium iodide (PI) [29] and 7-aminoactinomycin D (7-AAD) [30], which enters exclusively damaged cells, was used to determine cell death. For fluorescence-activated cell sorter (FACS) analysis, NIT-1, βTC, and αTC1 cells as well as dispersed islets were stained with PI (50 µg/mL, Sigma Immunochemicals) and dispersed islets were stained with7-AAD (50 µg/mL, BioLegend, Inc.). Briefly, 5 µL of PI or 5 µL of 7-AAD were added to 300 µL of cell suspension (5×10^5^ cells), gently mixed and incubated for 15 min at room temperature in the dark; and the cells were then immediately analyzed by flow cytometry on a FACSCalibur (Becton Dickinson, Oxford, United Kingdom) equipped with a single argon ion laser emitting an excitation light at 488 nm wavelength. Data on 10,000 cells were collected at a low flow rate and subsequently analyzed using CellQuest software.

### Electron Microscopic Studies for Isolated Islets and Islet Grafts

Labeled and unlabeled islets as well as islet grafts were fixed in 2.5% glutaraldehyde and postfixed in 1% osmium tetraoxide. They were then dehydrated using alcohol and embedded in epoxy resin for sectioning. Ultra-thin sections were stained with uranyl acetate and lead citrate, and examined with a Hitachi H-7500 transmission electron microscope (TEM). To determine elementary iron in the graft, we used electron energy-loss spectroscopy with JEOL 2010F (JEOL Ltd., Tokyo, Japan), 200 KeV, equipped with a Schottky-type emission gun and a Gatan Image Filter).

### Islet Transplantation

Three hundred C57BL/6 mouse islets cultured with and without CSPIO were transplanted under the left kidney capsule of each inbred C57BL/6 or Balb/c mouse. In a separate set of experiments, 200, 300 and 400 CSPIO-labeled C57BL/6 mouse islets were syngeneically transplanted under the kidney capsule of each inbred C57BL/6 mouse. (Table 1) To accomplish this, the islets were centrifuged in PE-50 tubing (Clay Adams, Parsippany, NJ) connected to a 200-µL pipette tip. With the mouse under amobarbital anesthesia, the left kidney was exposed through a lumbar incision. A capsulotomy was performed in the lower pole of the kidney, and the tip of the tubing advanced under the capsule of the upper pole, the site of final injection. The capsulotomy was left unsutured [26], [27].

**Table 1:** Summary of mouse islet transplantation for magnetic resonance imaging.

islet transplantation	recipients (number)	islet number	follow-up period
syngeneic	non-diabetic (14)	300	8 weeks
	non-diabetic (8)	200, 300, 400	5 weeks
allogeneic	non-diabetic (10)	300	31 days
	diabetic (4)	300	5 weeks

### In vivo MR Imaging of Transplanted Islets

Serial MR imaging of the recipients was performed every week or every other week from 1 day to 8 weeks after transplantation. Images were acquired on a 3.0 T MR scanner (Magnetom Trio with TIM, Siemens, Erlangen, Germany) using a home-made surface coil. Images from a heavy T2* weighted gradient-recalled echo sequence were acquired for all subjects [26], [27]. The quantification of the isograft MR signal intensity was done by using the contralateral kidney as a reference. Consecutive MR scans were performed in 2 sets of experiments: one at weeks 1, 3, and 5, and the other at weeks 2, 4, and 6 after transplantation.

### Removal of the Islet Graft

Eight weeks after transplantation, animals intended for graft removal were anesthetized with amobarbital. An abdominal incision was made and the kidney was exposed. Under a dissecting microscope, the kidney capsule surrounding the graft was excised and removed with the adherent graft [26], [27].

### Histological Study of the Islet Graft

The removed grafts were fixed in formalin solution and processed for paraffin embedding and sectioning. Sections of grafts were stained for iron with Prussian blue and for endocrine β-cells with a guinea pig anti-swine insulin antibody (Dako Co., Glostrup, Denmark) [26], [27].

### Statistical Analysis

Results were expressed as mean ± standard deviation (SD). Unpaired Student’s t test was employed to compare two groups. A value of p<0.05 was considered significant.

## Results

### Uptake of CSPIO by Isolated Islets

**Figure 1:**
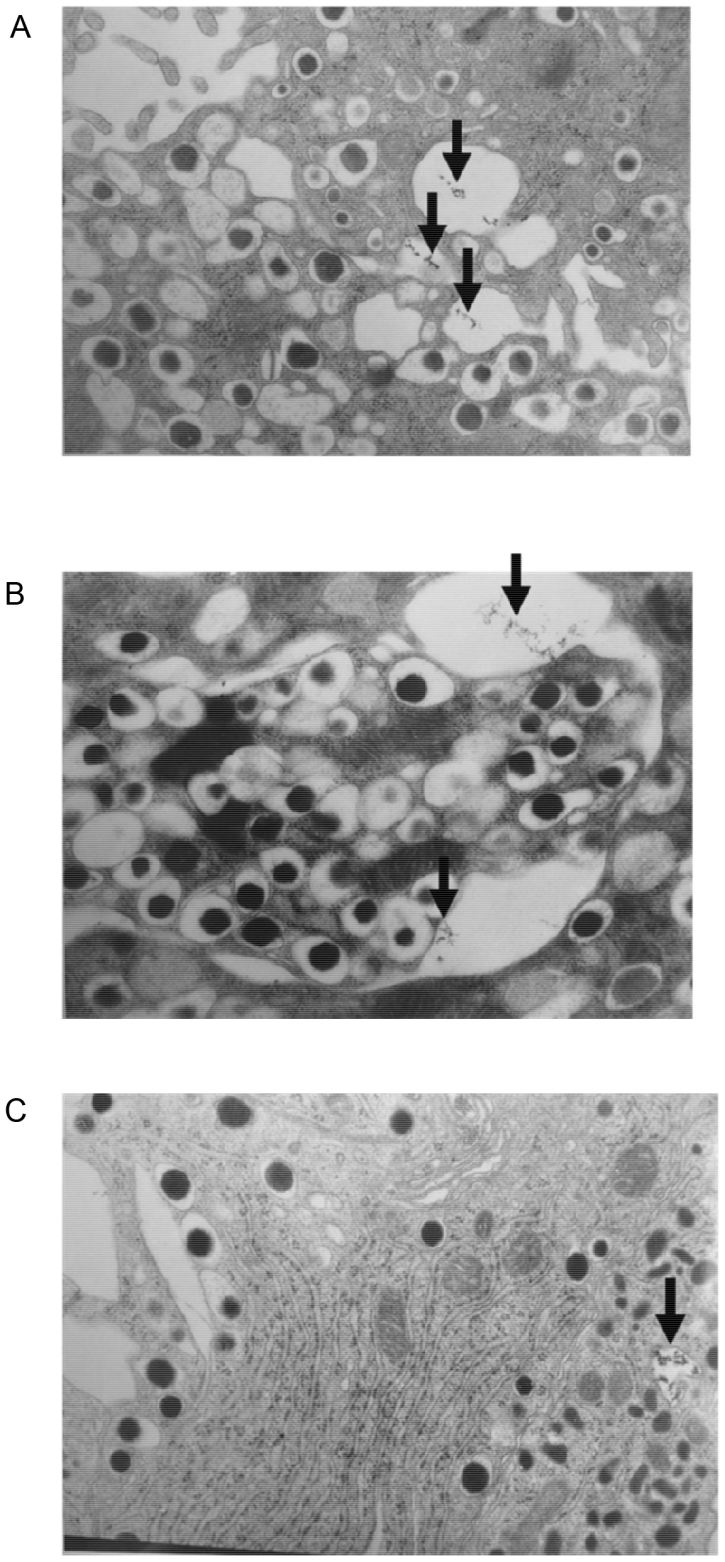
TEM micrographs of isolated islets incubated with CSPIO. At 1 h (A; ×10,000) and 4 h (B; ×10,000) after incubation, TEM showed CSPIO particles (indicated by the arrow) were within islets but between cells. However, CSPIO particles were observed in endocytotic vesicles of an alpha-cell at 8 h (C; ×10,000).

By using TEM, we observed endocytosis of CSPIO particles in isolated islets. At 1 and 4 h after incubation of isolated islets with CSPIO, characteristic granular CSPIO particles were within islets, but between individual cells (**Fig. 1A and B**). However, at 8 h, CSPIO particles were in endocytotic vesicles of both α-cells (**Fig. 1C**) and β- cells which exhibited intact ultrastructure. In contrast, CSPIO particles were not observed in unlabeled control islets.

### The Influence of CSPIO on Insulin Secretion and Cell Death of Islets

**Figure 2:**
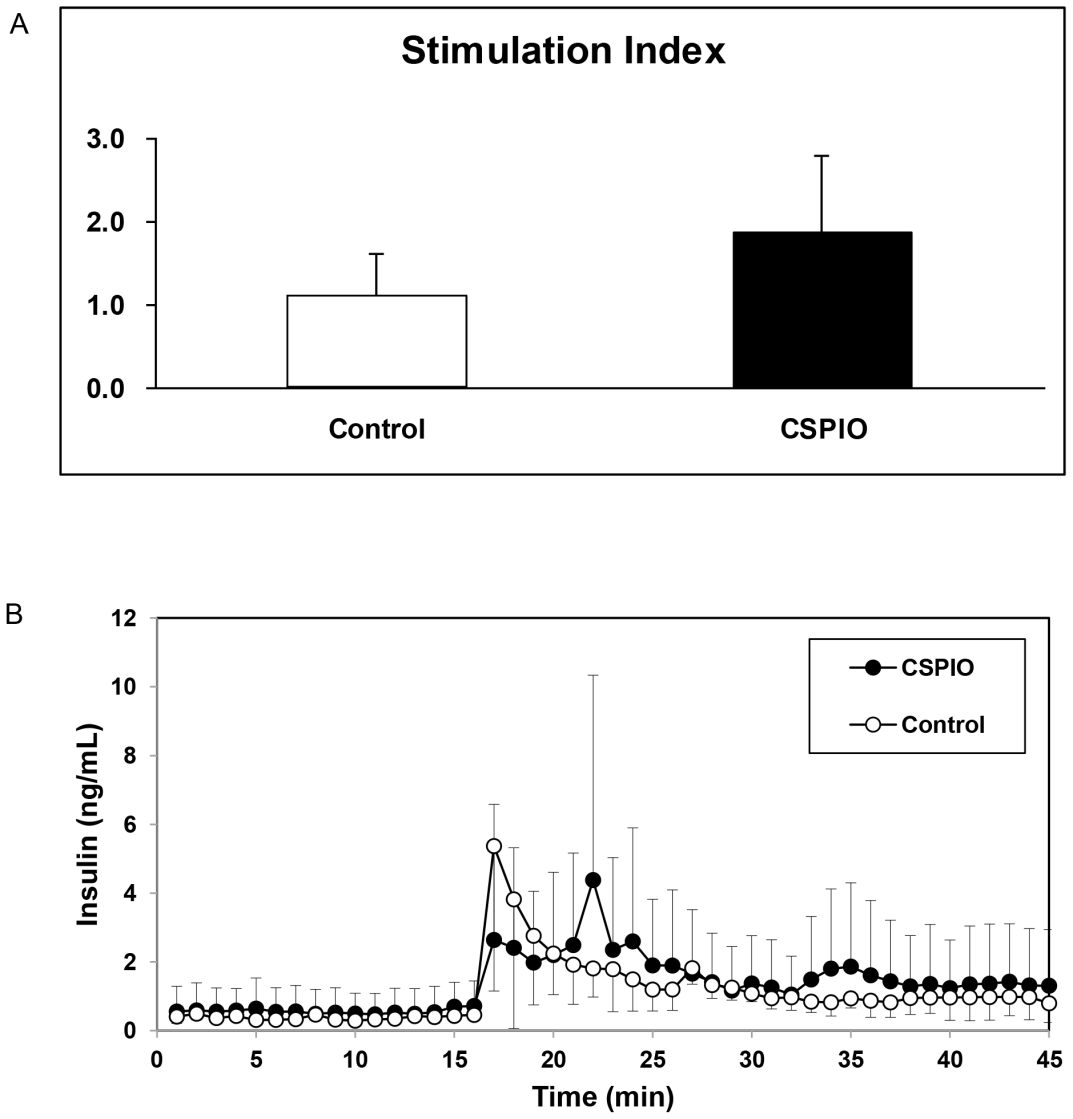
Insulin secretion of islets incubated with and without CSPIO. Studies of static incubation at 2.8 and 16.7 mmol/L glucose (A) and perifusion with 2.8 and 16.7 mmol/L glucose (B) showed the islets incubated overnight with [black column (n = 3) and solid circles (n = 3)] and without [white column (n = 3) and open circles (n = 3)] CSPIO (10 µg/mL) had comparable insulin responses to high glucose challenges. Stimulation indices were calculated by dividing the amount of insulin released with 16.7 mmol/L glucose by that released with 2.8 mmol/L glucose.

**Figure 3:**
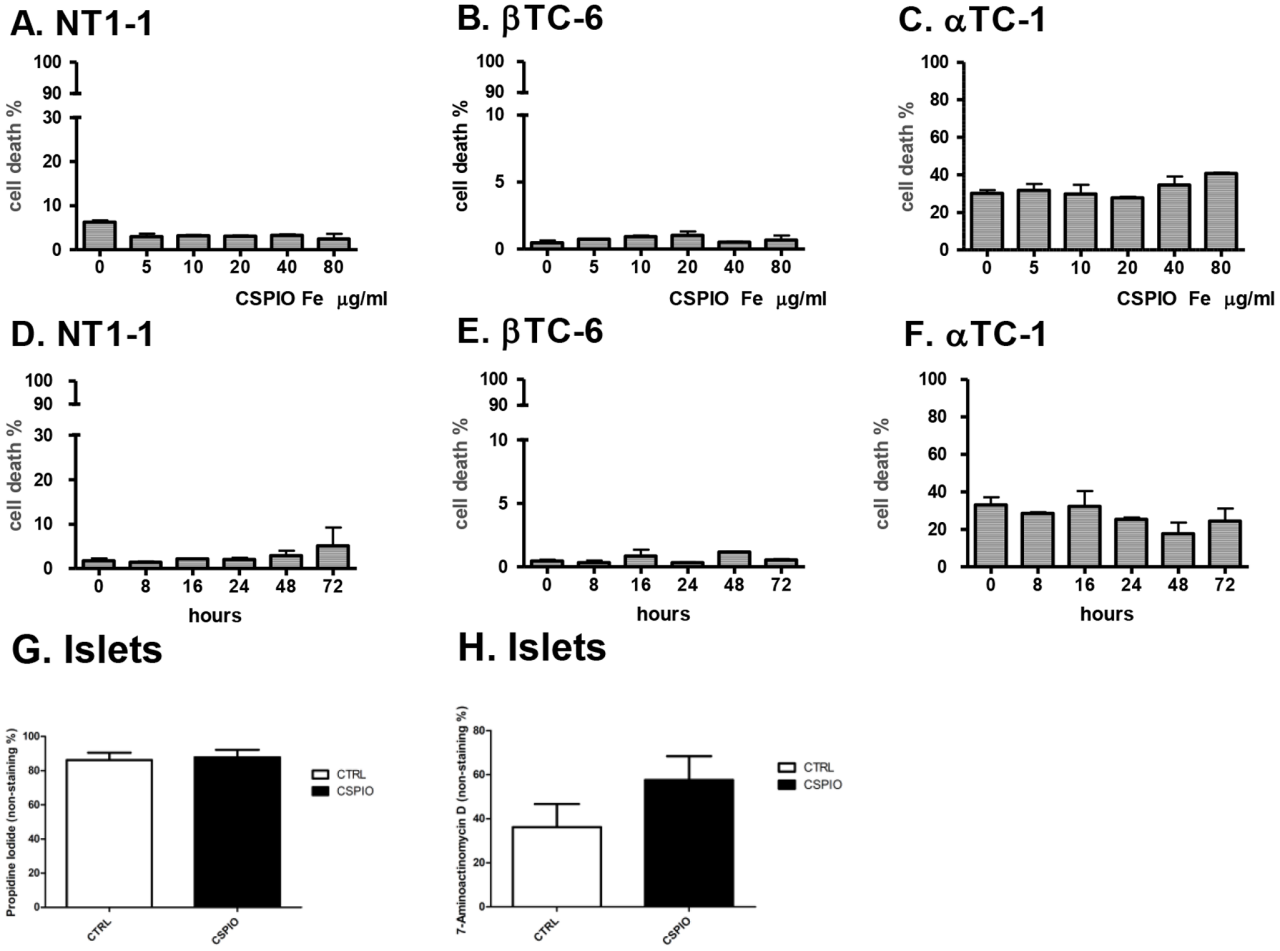
Death rates of NIT-1, βTC, and αTC1 cells as well as islets incubated with and without CSPIO. NIT-1 (A and D), βTC (B and E) and αTC1 (C and F) cells incubated with CSPIO did not increase death rates with increasing CSPIO concentrations up to 80 µg (A–C) or incubation time up to 72 h (D–F). In addition, overnight incubation of islets with CSPIO (10 µg/ml) did not affect cell viability (G, H). A–H were determined by propidium iodide (PI) and H by 7-aminoactinomycin D (7-AAD).

Islets that were overnight incubated with and without CSPIO had comparable stimulation indices (1.88±0.92 vs. 1.11±0.52, P = 1.0) with static incubation in 2.8 and 16.7 mmol/L glucose (**Fig. 2A**) During perifusion with sequential 2.8 and 16.7 mmol/L glucose, both labeled and unlabeled islets showed physiological first phase and second phase insulin secretion (**Fig. 2B**). Incubation of islets with CSPIO did not increase the death rates of NIT-1, βTC, and αTC1 cells with increasing CSPIO iron concentrations up to 80 µg/mL (**Fig. 3A–C**) or incubation time up to 72 h (**Fig. 3D–F**). Besides, overnight incubation of islets with 10 µg/ml CSPIO did not affect cell viability (**Fig. 3G, H**).

### Correlations between MR Images and Histological and Electromicroscopic Studies of CSPIO-labeled Islet Isografts

**Figure 4:**
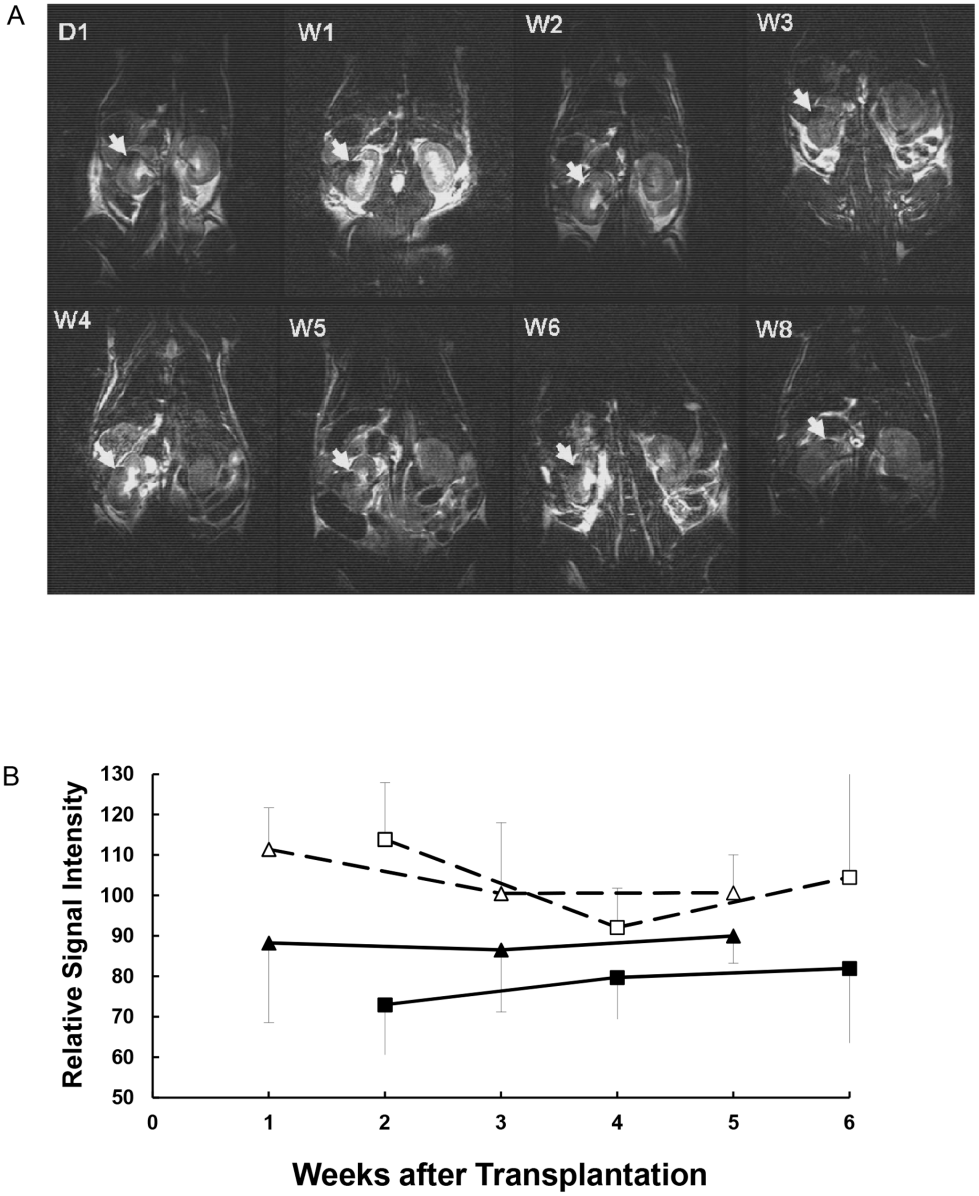
MR scans of CSPIO-labeled islet isografts in non-diabetic mice. A: Grafts of CSPIO-labeled islets were visualized on MR scans as distinct hypointense areas homogeneously located at the upper pole of the left kidney at day 1 and week 1, 2, 3, 4, 5, 6, and 8 after syngeneic transplantation in non-diabetic mice. B: Compared with the same area on the contralateral kidneys, the MR signal intensity in CSPIO-labeled (solid triangle and square) was significantly lower than that of control (open triangle and square) islet grafts (81.9±14.0% vs. 103.8±15.4%, P = 3.68297E-05).

**Figure 5:**
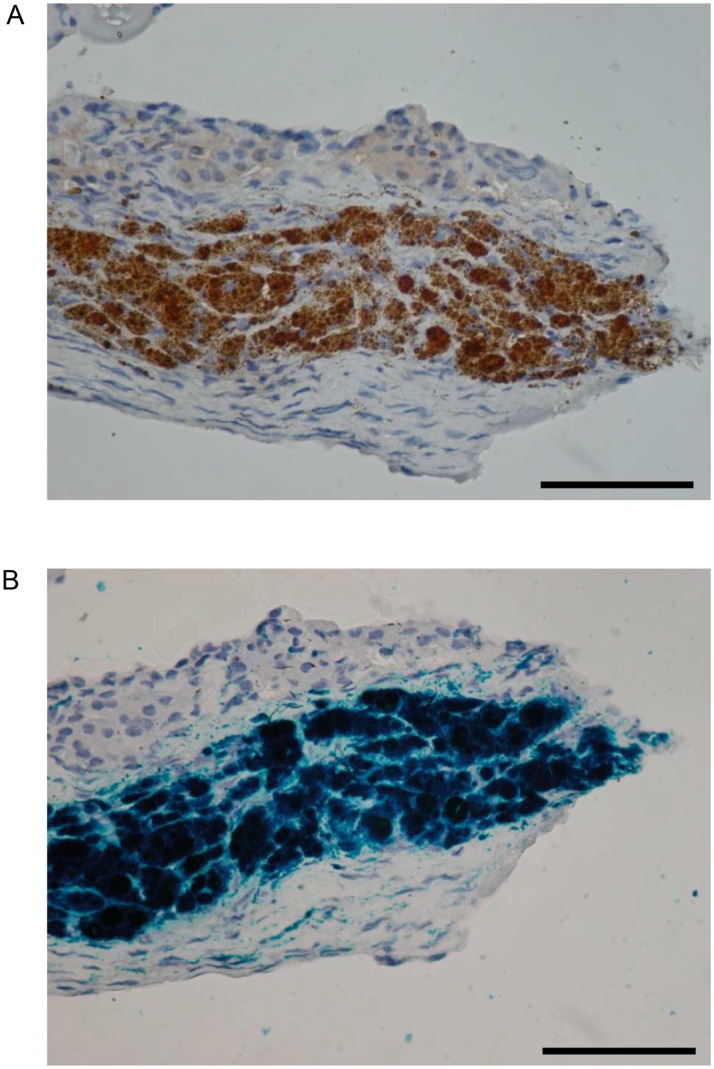
Histology of CSPIO-labeled islet isografts at 8 weeks after transplantation in non-diabetic mice. Colocalization of insulin (A; brown) and iron (B; blue) staining in CSPIO-labeled islet grafts. Scale bar: 100 µm.

**Figure 6:**
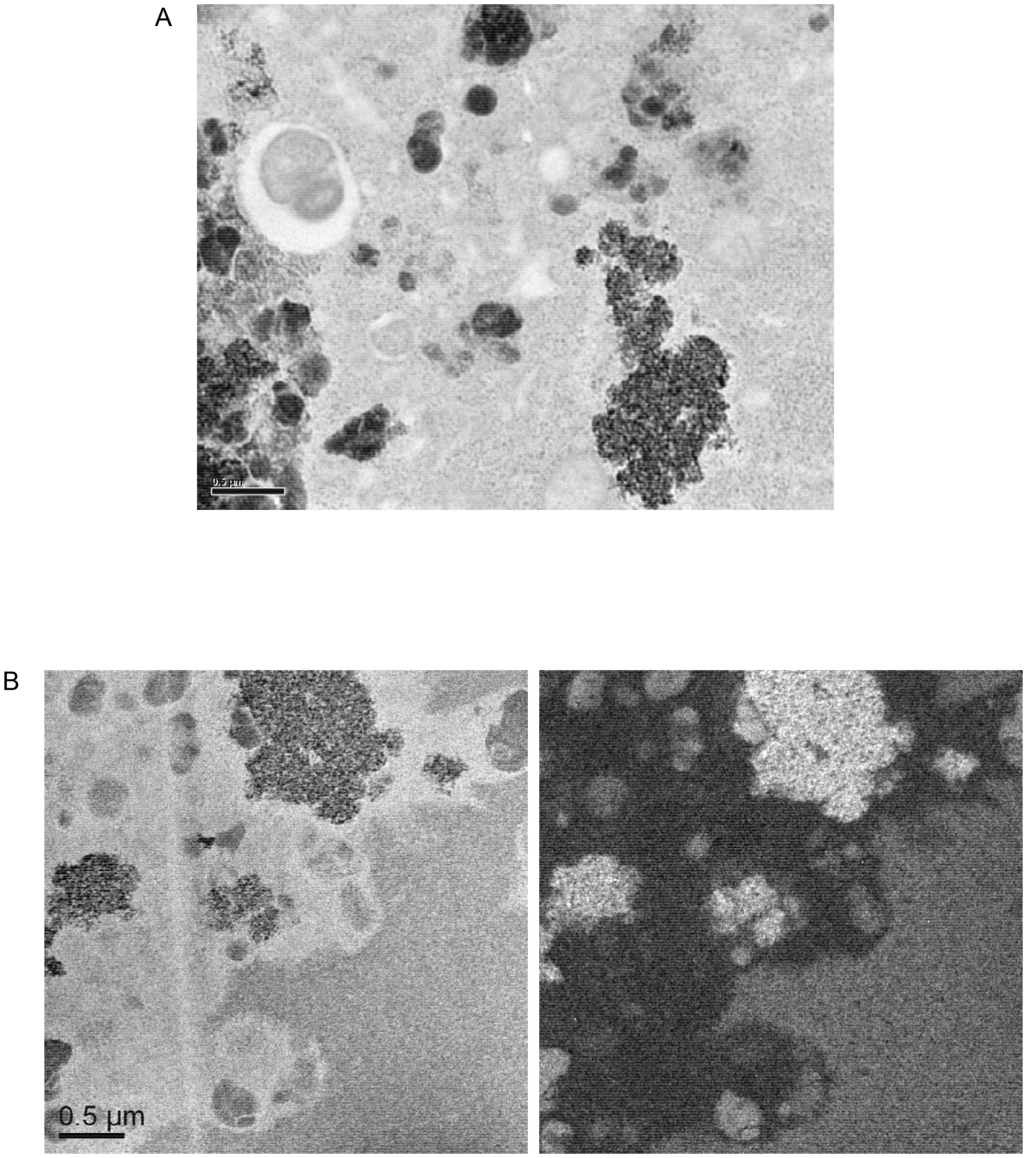
TEM micrographs and iron mapping of CSPIO-labeled islet isografts at 8 weeks after transplantation in non-diabetic mice. A: TEM showed several electron dense clumps distributed in the cytoplasm of a β-cell with intact ultrastructure. B: Electron energy-loss spectroscopy mapping of iron demonstrated significant signals of Fe recorded as bright areas in the islet graft.

From day 1 to 8 weeks after syngeneic islet transplantation in non-diabetic mice, grafts of CSPIO-labeled islets were visualized on MR scans as distinct hypointense areas homogeneously located at the upper pole of the left kidney, the site of transplantation. (**Fig. 4A**) The isograft MR signal intensity was quantified in 2 sets of experiments, one at weeks 1, 3, and 5, and the other at week 2, 4, and 6 after transplantation. The MR signal intensity of CSPIO-labeled and control islet isografts was 81.9±14.0% and 103.8±15.4%, respectively (P = 3.68297E-05, **Fig. 4B**). At 8 weeks after transplantation, the presence of CSPIO-labeled islet grafts under the kidney capsule was confirmed by the histological staining for insulin and Prussian blue, which showed colocalization of positive insulin and iron staining in the graft (**Fig. 5A and B**). Under TEM, there were several electron dense clumps distributed in the cytoplasm of islets that exhibited intact ultrastructure (**Fig. 6A**). Electron energy-loss spectroscopy further demonstrated that these clumps contained elementary iron (**Fig. 6B**).

In a separate set of experiments, we syngeneically transplanted 200, 300, and 400 CSPIO-labeled islets under the mouse kidney capsule, and found that their MR images showed similar signal loss in grafted kidneys.

### Correlations between the MR Images and Histological Studies of Islet Allografts

**Figure 7:**
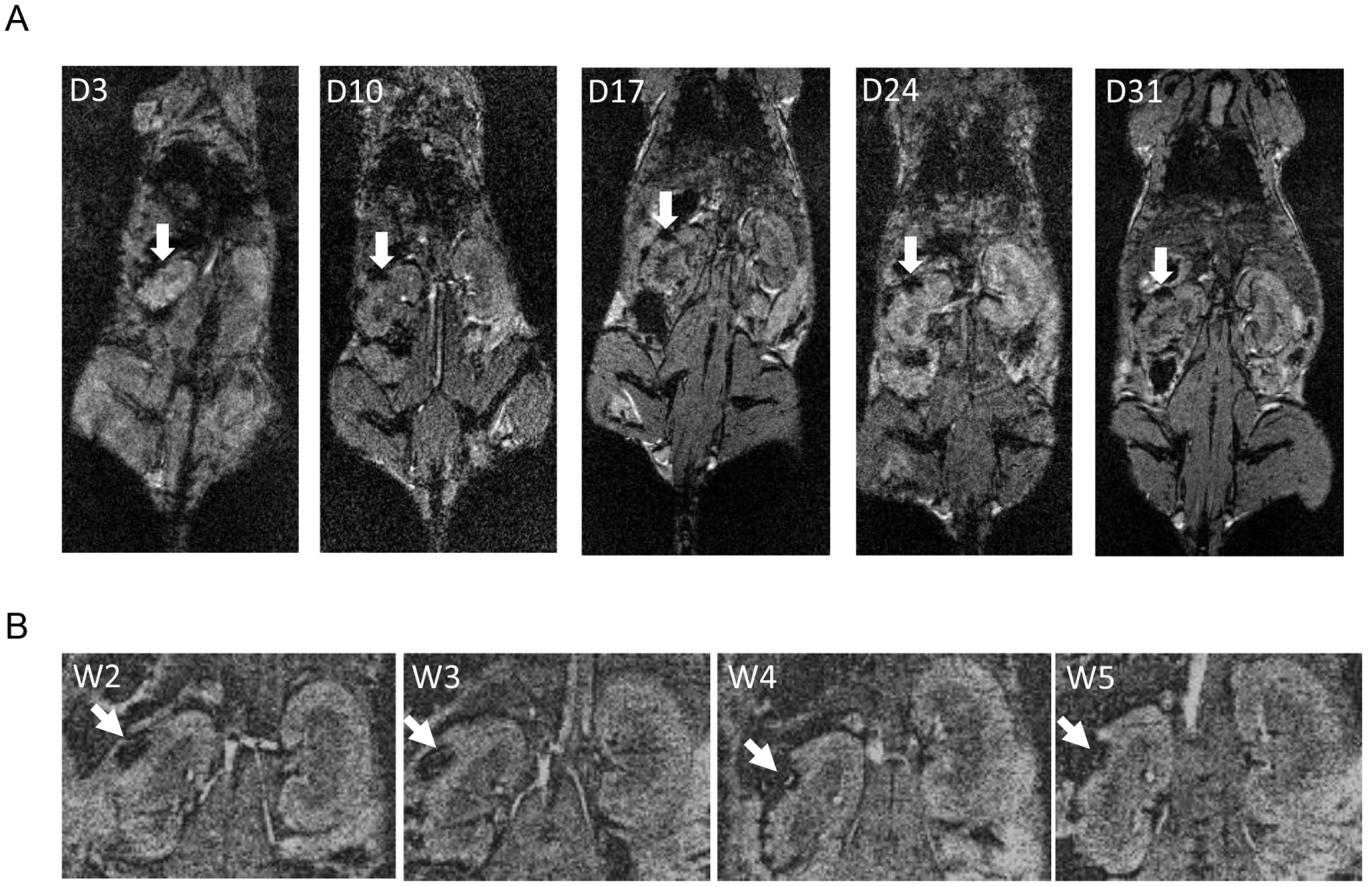
MR scans of CSPIO-labeled islet allgrafts in a non-diabetic (A) and a diabetic (B) mouse. (**A**) At day 3, 10, 17, 24, and 31 post-allotransplantation in a non-diabetic recipient, MR scans showed hypointense areas at the upper pole of the left kidney. (**B**) In a streptozotocin-induced diabetic recipient, there was no significant change of MR hypo-intensity at the upper pole of left kidney for 2–5 wks although its blood glucose levels decreased in the first 2 weeks and then went up. Our MR images could not differentiate the signal loss in grafted kidneys between the diabetic and non-diabetic mouse.

**Figure 8:**
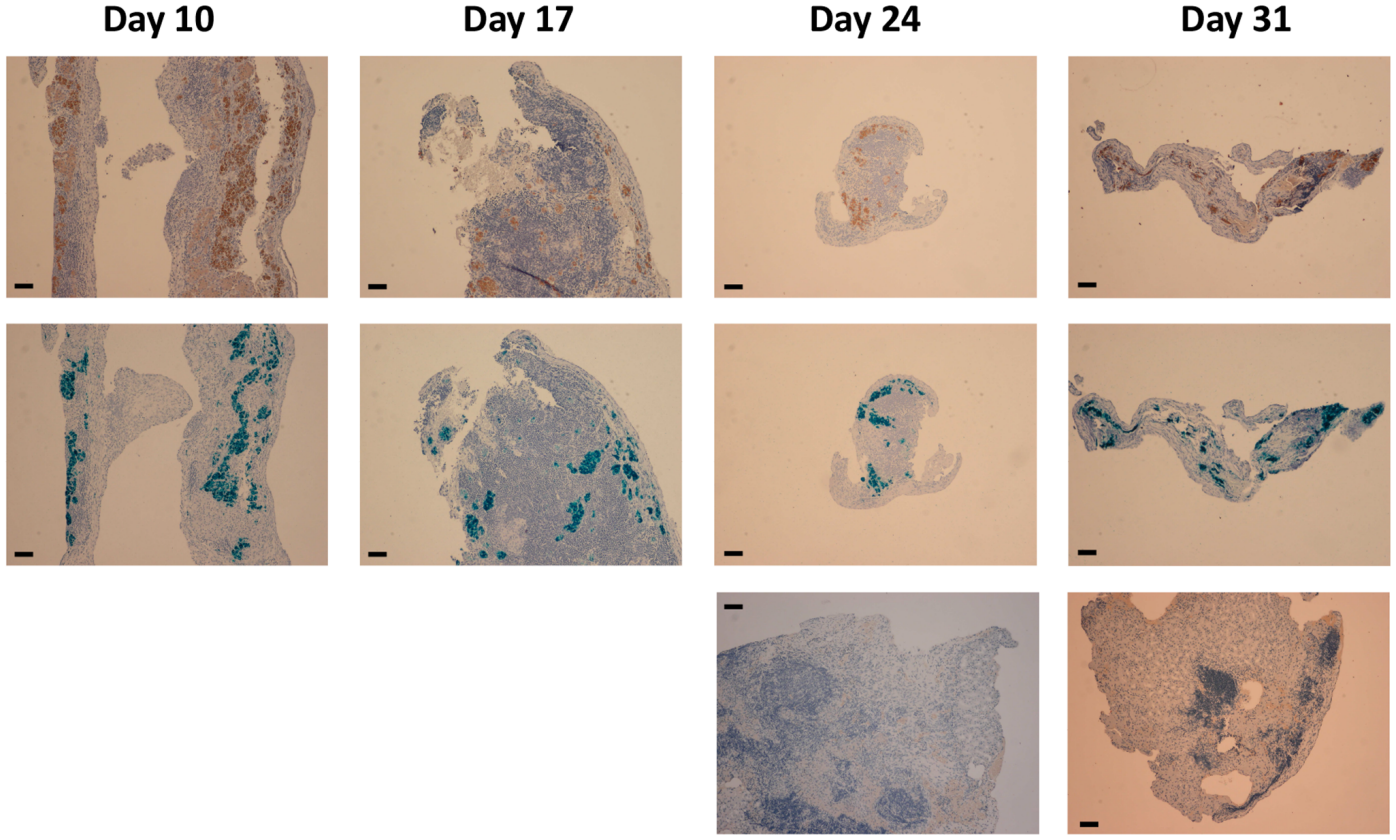
Histology of islet allografts at day 10, 17, 24, and 31 after transplantation in non-diabetic mice. Upper row: insulin staining of CSPIO-labeled islet grafts; middle row: Prussian blue staining of CSPIO-labeled islet grafts; lower row: insulin staining of unlabeled islet grafts. There was profound immune cell infiltration at day 10 and 17, and a decrease in graft size at day 24 and 31 in the CSPIO-labeled islet graft. However, colocalization of insulin staining (shown in brown) and iron staining (shown in blue) presented in the CSPIO-labeled islet graft all the time. In contrast, the unlabeled islet graft at day 24 and 31 contained many immune cells but no insulin-positive cells. Magnification: 100×; Scale bar: 100 µm.

At day 3, 10, 17, 24, and 31 after allotransplantation of CSPIO-labeled islets in non-diabetic mice, MR scans showed persistent hypointense areas at the upper pole of the left kidney (**Fig. 7A**). In addition, we also allotransplanted 300 CSPIO-labeled islets under the kidney capsule of each streptozotocin-induced diabetic mouse. There was no significant change of MR hypo-intensity at the upper pole of left kidney from 2 to 5 wks (**Fig. 7B**) although recipients’ blood glucose levels decreased in the first 2 weeks and then went up. Moreover, our MR images could not differentiate the signal loss in grafted kidneys between diabetic and non-diabetic mice (**Fig. 7A and B**). We examined the histology of CSPIO-labeled islet grafts in 2 mice at day 10, 17 and 24, respectively, and 4 mice at day 31. In CSPIO-labeled and unlabeled islet grafts, there was profound infiltration of immune cells, and a progressive decrease in graft size with time. However, colocalization of insulin and iron staining presented in the CSPIO-labeled grafts all the time, in contrast to the disappearance of insulin-positive cells in the unlabeled islet graft at day 24 and 31 (**Fig. 8**).

## Discussion

MRI has shown promising results in the study of insulitis, pancreatic β-cell mass, and islet transplantation in animal models. (31) Recently, Gaglia et al. showed that MR imaging of infused ferumoxtran-10 nanoparticles permitted effective visualization of the islet inflammation and distinction of recent-onset diabetic patients from non-diabetic controls. (32) Ferumoxtran-10 has a size of 30 nm (33) with favorable biodistribution (34) which allows it to extravasate from the leakage vessels into the surrounding tissue and is engulfed by infiltrating cells, particularly macrophages. In contrast, anionic iron oxide nanoparticles are suitable for cellular labeling due to a high cellular uptake (34). Therefore, in the present study, CSPIO with a size of 87.2 nm (25) was taken up by the islets prior to transplantation. On T2*-weighted MR images, the labeled islets were clearly visualized as hypointense areas at the upper pole of the left kidney of both diabetic and nondiabetic mice for a long period.

In this study, we showed that CSPIO nanoparticles, a novel MR contrast agent, were taken up by islets in vitro, and CSPIO-labeled islet grafts under the kidney capsule could be visualized by MR imaging after syngeneic and allogeneic transplantation in mice. These findings are consistent with previous reports of the islet graft imaging at this site by using dextran-coated SPIO, including ferumoxide (Feridex®, Endorem™) and ferucarbotran (Resovist®) [8]–[11]. Therefore, our newly developed CSPIO nanoparticles are potentially applicable as a contrast agent for clinical islet imaging after manufacturing cessation of Feridex® and Resovist® [23].

Our electron microscopic studies revealed that endocytosis of CSPIO occurred at 1 h after incubation of isolated islets with CSPIO nanoparticles. Next, CSPIO was within islets but between individual cells at 1 and 4 h, and then at 8 h, in endocytotic vesicles of both α- and β-cells with intact ultrastructure. In contrast, we found Feridex® was in β-cells as early as 1 h after its incubation with isolated islets (data not shown). Previously, Berkova et al. showed Resovist® particles in endocytic structures of macrophages after 1-h labeling and then localized in β-cell vesicles at 4 h [16]. Taken together, different SPIO particles have variable uptake characteristics, and the uptake of CSPIO by islets is slower than that of Feridex® and Resovist®. Presumably, being cultured with islets, SPIO particles may localize in any islet cell types including α-, β-, δ-, PP-cells and islet macrophages. [10], [16] However, in this study, CSPIO was observed within α- and β-cells because they are two major populations in the islet.

An earlier study reported Resovist®-labeled rat islets lost 53% of their glucose-stimulated insulin secretion (GSIS) compare to unlabeled islets [12]. But later, the same group showed oppositely that in vitro GSIS was not affected by Resovist® labeling [13], [14]. In addition, rat [15], neonatal pig [15] and human [8], [9], [35] islets labeled with and without Feridex® had comparable GSIS. In this study, we also demonstrated that CSPIO labeling did not compromise insulin secretion of mouse islets. In addition to static incubation used in above-mentioned investigations, we perifused islets with low and high concentrations of glucose and showed that CSPIO-labeled mouse islets maintained their physiological first and second phase insulin secretion as shown in unlabeled islets. Regarding the safety of labeling islets with SPIO, it has been shown that islet viability was not disrupted by the Feridex® labeling procedure up to 48 h [9], [16], [35] and there was no increased apoptosis in islets after 24-h culture with Feridex® [9], [10], [35]. Recently, we found that the cell death rates in RAW cells incubated with CSPIO did not increase with increasing CSPIO concentrations or longer incubation time [26]. Here, we further examined the cytotoxicity of CSPIO on α- (αTC1) and β-cell (NIT-1 and βTC) lines as well as islets with the same results. Therefore, mouse islets labeled with CSPIO maintain their viability and insulin secretory function, which are feasible for in vivo MR imaging.

Previously, Evgenov et al. implanted 1,000 human islets labeled with dextran-coated SPIO under the left mouse kidney capsule, and unlabeled islets under the right kidney capsule. They observed a 20% decrease in 4.7 T MR T2 values at the left kidney in comparison to the right kidney, for up to 188 days [9]. Tai JH et al. also showed 1.5 T MR signal loss of rat isografts of 200 Feridex®-labeled islets under the kidney capsule was 64.4% at week 1 and 68.8% at week 5 in comparison to background tissue [15]. Recently, we used a 3.0 T MR scanner and detected CSPIO-labeled islet isografts in 2 mice as a distinct homogeneously hypointense area persistently located at the upper pole of the left kidney for up to18 weeks. Using the contralateral kidney (without transplant) as a reference, the signal loss was 60% compared to that of one control mouse [26]. In the present study, we increased the mouse numbers and found that the signal loss was 20% lower in CSPIO-labeled islet isografts than that of unlabeled islet isografts, and this difference persisted for 6 weeks. Therefore, our CSPIO is as effective as dextran-coated SPIO for MR tracking islet grafts under the kidney capsule. In the future, we need to investigate the application of CSPIO at the intrahepatic site where MR imaging has shown excellent visualization of islet grafts labeled with dextran-coated SPIO [8], [9], [10], [12], [14], [16]–[22], [35], [36].

By using 1.5 T MR scan, Tai et al. detected as few as 200 Feridex®-labeled islet isografts under the rat kidney capsule [15]. In the present study, our technique showed comparable sensitivity, and detected isografts of 200 islets labeled with CSPIO at the same transplant site. Previously, Evgenov et al. demonstrated that 1,000 Feridex®-labeled human islets transplanted into livers of diabetic NOD.scid mice had a higher rate of islet death than non-diabetic mice on in vivo MR images [10]. However, our MR images could not differentiate between signal loss in grafted kidneys from diabetic versus non-diabetic mice transplanted with 300 CSPIO-labeled islets. Presumably, larger numbers of islets and the intrahepatic site are favorable for quantification of MR images.

Our histological analysis of CSPIO-labeled islet isografts removed at 8 weeks after transplantation showed the colocalization of insulin and iron staining in the same areas, which is consistent with previous reports involving Feridex®-labeled islet isografts at 36 days [15]. Using TEM, we demonstrated for the first time that electron dense clumps in the cytoplasm of islet cells that exhibited intact ultrastructure. It was further proved by using the electron energy-loss spectroscopy that these clumps in islet cells contained elementary iron. Our findings indicate that CSPIO nanoparticles exist in islet cells but do not alter their ultrastructure for up to 8 weeks after syngeneic islet transplantation.

Following intraportal allotransplantation of Feridex®- and Resovist®-labeled islets without immunosuppression, Kriz et al. observed that the number of hypointense spots on MR scans gradually decreased in diabetic rats and mice from the second week, while the number declined only insignificantly in the syngeneic group [14], [36]. Histologically, they found that allogeneic islets were completely rejected, and iron particles in macrophages were detected in the syngeneic islets but were absent in the rejected islet structures. In contrast, our MR scans of CSPIO-labeled islet allografts under the kidney capsule showed persistent hypointense areas from day 3 to 31. Although the histology revealed that CSPIO-labeled allografts gradually decreased in size with profound infiltration of immune cells, colocalization of insulin and iron staining presented all the time. Prolonged existence of our CSPIO-labeled islet allografts may be due to different transplantation sites and different SPIO nanoparticles. The implanted islets are concentrated under the kidney capsule but dispersed in the liver; thus, the former may maintain longer positive MR images and more residual cells during allorejection. Besides, the SPIO particles may be metabolized faster in the liver where they are sequestered by Kupffer cells [37]. Moreover, the coating materials of CSPIO and Feridex®/Resovist® may also influence the clearance rate of their iron oxide [38]. In contrast to CSPIO-labeled islet grafts, unlabeled islet grafts at day 24 and 31 contained no insulin-positive cells but many immune cells. Whether CSPIO protected islet allografts from immune rejection needs to be further elucidated.

In this study, we demonstrated that our newly developed CSPIO nanoparticles were taken up by islets in vitro, did not affect insulin secretion and death rates of islets, and could be visualized by MR imaging after syngeneic and allogeneic transplantation of CSPIO-labeled islets under the kidney capsule in mice. These results indicate that CSPIO is a potential contrast agent for MR imaging of islet grafts.
